# *Salmonella* Typhimurium TTSS-2 deficient mig-14 mutant shows attenuation in immunocompromised mice and offers protection against wild-type *Salmonella* Typhimurium infection

**DOI:** 10.1186/1471-2180-13-236

**Published:** 2013-10-22

**Authors:** Niladri Bhusan Pati, Vikalp Vishwakarma, Sathish kumar Selvaraj, Sabyasachi Dash, Bhaskar Saha, Neera Singh, Mrutyunjay Suar

**Affiliations:** 1 School of Biotechnology, KIIT University, Bhubaneswar 751024, Odisha, India; 2 National Centre for Cell Science, Ganeshkhind, Pune, India

## Abstract

**Background:**

Development of *Salmonella enterica* serovar Typhimurium (*S.* Typhimurium) live attenuated vaccine carrier strain to prevent enteric infections has been a subject of intensive study. Several mutants of *S*. Typhimurium have been proposed as an effective live attenuated vaccine strain. Unfortunately, many such mutant strains failed to successfully complete the clinical trials as they were suboptimal in delivering effective safety and immunogenicity. However, it remained unclear, whether the existing live attenuated *S*. Typhimurium strains can further be attenuated with improved safety and immune efficacy or not.

**Results:**

We deleted a specific non-SPI (Salmonella Pathogenicity Island) encoded virulence factor *mig-14* (an antimicrobial peptide resistant protein) in *ssaV* deficient *S.* Typhimurium strain. The *ssaV* is an important SPI-II gene involved in *Salmonella* replication in macrophages and its mutant strain is considered as a potential live attenuated strain. However, fatal systemic infection was previously reported in immunocompromised mice like *Nos2*^−/−^ and *Il-10*^−/−^ when infected with *ssaV* deficient *S*. Typhimurium. Here we reported that attenuation of *S*. Typhimurium *ssaV* mutant in immunocompromised mice can further be improved by introducing additional deletion of gene *mig-14*. The *ssaV, mig-14* double mutant was as efficient as *ssaV* mutant, with respect to host colonization and eliciting *Salmonella*-specific mucosal sIgA and serum IgG response in wild-type C57BL/6 mice. Interestingly, this double mutant did not show any systemic infection in immunocompromised mice.

**Conclusions:**

This study suggests that *ssaV, mig-14* double mutant strain can be effectively used as a potential vaccine candidate even in immunocompromised mice. Such attenuated vaccine strain could possibly used for expression of heterologous antigens and thus for development of a polyvalent vaccine strain.

## Background

Enteric infections represent a major threat to human health worldwide affecting both children and adults in developing and industrialized countries. These infections are caused by a number of pathogens including *Salmonella, Shigella, Campylobacter species*, *Aeromonas, Plesiomonas*, *Vibrio*, *Yersinia entercolitica*, *E. coli* 0157:H7 and *Rotavirus.* Among these enteric pathogens, *Salmonella enterica* with more than 2500 serovars is considered as a key pathogen that can infect a wide range of host species and is the leading cause of acute gastroenteritis. The increased mortality, morbidity and limited availability of specific drugs against these infection demands an alternative to reduce the global disease burden. One such promising alternative is the development of live-attenuated vaccines. These vaccines are attenuated forms of the pathogen itself which can provide defense against the infection from the same pathogen. In case of *Salmonella*, a facultative intracellular pathogen, specific cell mediated immune response is critical to control and clear the pathogen from the host [1-4]. In order to stimulate cellular immunity with higher efficacy, live attenuated *Salmonella* are preferred over the inactivated or killed vaccine candidates [5-7]. Ideally, a live attenuated vaccine strain should be able to withstand the host stress, provide defense against the concerned pathogen and should successfully colonize the host lymphoid tissues while retaining its avirulent nature. Researchers have established mice models in order to efficiently screen the possible vaccine attributes of genetically modified *Salmonella enterica* strains or their derivatives [8-12]. However, many live attenuated strains are known to develop systemic infection when administered to immune deficient individuals [13-15]. In order to prevent the systemic infection in immune-compromised patients, it is very crucial to attain sufficient attenuation. Many attenuated *Salmonella* vaccine strains carrying deletion mutation either in the metabolic gene or in the virulence factors have been developed but with a little success in the clinical trials [16]. This study primarily focuses on the development of an improved live-attenuated *S.* Typhimurium strain. A number of *S.* Typhimurium mutants developed, are known to elicit optimal immune response but showed reduced survival efficacy [17-26]. Earlier studies have shown that only a few such mutants have been actually tested in a pilot study in order to investigate their protection efficacy [27-29]. When tested, such a few proposed vaccine strains resulted in developing diseases in the hosts of variable immune status [20,30-32]. Therefore, the development of a safer immunogenic live-attenuated *S*. Typhimurium strain is a need of an hour [33] and can be accomplished by development of a suitably attenuated strain with an avirulent property in immunocompromised individuals. Previous studies have shown that TTSS-2 deficient *S.* Typhimurium strains were highly attenuated and conferred protection from further challenges of wild-type *S.* Typhimurium by eliciting O-antigen specific serum IgG and secretory IgA in C57BL/6 mice [34-36]. In a recent study, the *ssaV* mutant of *S.* Typhimurium was found to be virulent in immune compromised C57BL/6 mice devoid of *Nos2* and *Il-10* gene [37]. These two mice strains were used as they lack key elements of the antibacterial defense like the inducible nitric oxide (NO) synthase, a reactive oxygen species generating enzyme and interleukin-10 gene [38]. In this study, we have also used CD40L KO mice to screen the attenuation of proposed vaccine strain. This particular mouse model is used as it is partially immunocompromised in terms of generation of different class of antibodies.

Virulence of TTSS-2 deficient *S*. Typhimurium in immunocompromised mice unveils the role of other factors favoring the replication and long-term survival of *S*. Typhimurium in host tissues. Mig-14, an antimicrobial peptide resistance protein, is one such important factor that supports the long-term persistence of *Salmonella* in the macrophages [39]. Mig-14 protein binds to the anti-microbial peptides like CRAMPS to protect *Salmonella* from antimicrobial peptides [40]. The presence of Mig-14 in the periplasmic localization inhibits the entry of antimicrobial peptides to the cytoplasm of the bacterium, eventually making macrophage a good niche for *Salmonella* to replicate and survive. This study proposes a diverse role for mig*-14* in the survival of TTSS-2 deficient *Salmonella* in immunocompromised mice like *Nos2*^*−/−*^*, Il-10*^*−/−*^*and CD40L*^*−/−*^ and explores the possible potential of *S.* Typhimurium *ssaV* and *mig-14* double mutant as a safe vaccine carrier strain.

## Methods

### Bacterial strains and plasmids

Streptomycin resistant *S.* Typhimurium SB300 and *Salmonella* Enteritidis P125109 (*S*. Enteritidis) strains were taken as the wild-type controls [41,42]. Mutants MT5 (SB300; Δ*ssaV*) and MT4 (SB300; Δ*ssaV*, Δ*mig-14*) were generated by lambda red-mediated recombinase process [43]. Briefly, the host bacterial strain to be mutated was transformed with plasmid pKD46 and induced with arabinose (10 mM). The kanamycin open reading frame was PCR-amplified from template plasmid pKD4 using gene specific knockout primers (Table 1). The cassette was introduced into host bacterial genome with the help of Exo, Bet and Gam proteins from induced pKD46 plasmid of host bacterial strain. The positive mutants were selected on LB agar plates supplemented with kanamycin (50 μg/ml) and mutation in the target gene was confirmed using gene specific confirmatory primers in combination with respective forward knock-out primer (Table 1). Later, the antibiotic cassette was flipped by plasmid pCP20 [43]. An ampicillin resistant plasmid (pM973) was used to maintain the ampicillin resistant trait in wild-type strain (SB300) while challenging vaccinated mice groups with wild-type *S*. Typhimurium [44]. The bacterial strains and the plasmids used in this study are listed in Table 2.

**Table 1:** Primers used in the study

	
Fw-ssaV	AGT CGC AAT GCG TTC ATG GTT AG
Rw-ssaV	TTC TTC ATT GTC CGC CAA CTC
KO-Fw-ssav	AAT AAA ATT TCT GGA GTC GCA ATG CGT TCA TGG TTA GGT GAG GGA TGT GTA GGC TGG AGC TGC TT
KO-Rw-ssaV	GCA TCA ATT CAT TCT TCA TTG TCC GCC AAC TCC TCT TCG CTA AGG ATA TGA ATA TCC TCC TTA GT
Conf-ssaV	GCA AAG CTT TGC TGC CAT TAA TCC
Fw-mig14	GAG TTT TGG TGA AAA TAC AAG AAG
Rw-mig14	GTA TAG TGT AAG TGA ATT TCG AGT AAT TG
KO-Fw-mig14	AGC AAA AAA ATA ATA CAA AAT AGC ATT TTC AGT AAG CTA AGT CAG TGT GTA GGC TGG AGC TGC TT
KO-Rw-mig14	GAA AAA TCT GGA CGT AAA AAA CAT ATT TAC GTC CAG GCT TTC TTT ATA TGA ATA TCC TCC TTA GT
Conf-mig14	CAT CAT CTG TTC CTG ACG CCA G

**Table 2:** Bacterial strains and plasmids used in the study

**Strains**	**Genetic information**	**Background**	**References**
SB300	*Salmonella* Typhimurium, *Sm*^*r*^	Wild type	[41]
M1525	*Salmonella* Enteritidis 125109 wild type; *Sm*^*r*^	Wild type	[42]
MT4	*S.* Typhimurium Δ*ssaV,*Δ*mig-14; Sm*^*r*^	SB300	This study
MT5	*S.* Typhimurium Δ*ssaV*; *Sm*^*r*^	SB300	This study
**Plasmids**	**Relevant genotype (S) and/or phenotype (S)**	**Resistance**	**References**
pM973	*bla* P*ssaH* gfpmut2 plasmid with *ori*pMB1	Amp^r^	[44]
pKD46	Red recombinase expression plasmid; P_araB_; oriR101	Amp^r^	[43]
pKD4	Template plasmid; FRT-*aphT*-FRT	Km^r^	[43]
pCP20	FLP recombinase expression plasmid	Cm^r^, Amp^r^	[43]

### Bacterial growth condition

Luria-Bertani medium supplemented with 0.3 M sodium chloride (SPI-1 inducing medium) was used to grow all the bacterial strains (Table 2) at 37°C for 12 h. Strains were diluted 1:20 in fresh SPI-1 inducing medium and sub-cultured for another 4 h until the bacteria attained their early log phase. Bacterial cells were pelleted, washed in ice-cold phosphate buffered saline (PBS) and approximately 5 × 10^7^ CFU were suspended in 50 μl cold PBS for use in the *in vivo* experiments. All the strains were tested for growth attenuation for 16 h in 10 ml of culture medium at 37°C with 150 rpm under aerated conditions.

### Ethical statement

All the animal experiments were performed in strict accordance with guidelines laid by the Institutional Animal Ethics Committee (IAEC) of National Centre for Cell Science (NCCS) Pune, India; Permit Number: 7/1999/CPCSEA-09/03/1999.

### Mouse lines

All experimental mice were specific pathogen free (SPF) C57BL/6 maintained in individually ventilated cages (IVC) (Tacket *et al.,* 1992). Wild-type, *Nos2*^−/−^ (B6.129P2- *Nos2tm1Lau*/J), *Il-10*^*−/−*^ (B6.129P2-Il10^tm1cgn^/J) *and CD40L*^*−/−*^ (B6.129S2-Cd40lg^tm1Imx^/J) mice were procured from Jackson Labs (Bar Harbor, ME) and bred in the C57BL/6 background at the animal facility of National Center for Cell Sciences (NCCS), Pune, India.

### Mice infection experiment for assessment of strain attenuation

The infection experiments were performed in streptomycin pretreated SPF mice in IVC as described earlier [45,46]. C57BL/6, *iNos*^−/−^, *Il10*^*−/−*^*and CD40L*^*−/−*^ mice were pretreated orally with 50 mg of streptomycin before infecting with wild-type and mutant strains. After 24 h, mice were infected with 5 × 10^7^ CFU (oral gavage) of the corresponding bacterial strain (i.e. MT5, MT4 and SB300). The bacterial load in the cecum, mesenteric lymph nodes (mLNs), liver and spleen was determined by plating the respective tissue homogenates on MacConkey agar plates supplemented with appropriate antibiotics (Streptomycin, 50 μg/ml; kanamycin, 50 μg/ml; ampicillin, 100 μg/ml). For statistical analysis, samples without bacterial counts were adjusted to the minimum detection level (10 CFU/organ in the mLN, 20 CFU/organ in the spleen, 10/x CFU/g, where x represents the net weight of the cecum content or feces collected). Cecal pathology of the infected mice was scored to analyze the degree of inflammation [45].

### Histopathological evaluation

Segments of the cecum, colon and ileum were embedded in Optimum Cutting Temperature solution O.C.T. (Sakura Finetek Inc., USA), snap-frozen in liquid nitrogen, and stored at −80°C. The 5 μm thick tissue sections were obtained on glass slides and stained with hematoxylin and eosin (H&E) stains after drying for at least 2 h at room temperature. The stained cryosections were evaluated on the basis of a previously described scoring system for the quantitative analysis of cecal inflammation [45,47]. The sections were scored on the basis of the pathological changes that include sub-mucosal edema (0–3), polymorphonuclear leukocyte infiltration (0–4), loss of goblet cells (0–3) and epithelial ulceration (0–3). The cumulative pathological scores ranged from 0 to 13 with arbitrary units covering the inflammation levels that included intact intestine without any sign of inflammation (pathoscore 0); minimal sign of inflammation (pathoscore 1–2), which is commonly found in the ceca of specific pathogen-free mice and generally not considered as a pathological feature; slight inflammation as a minimal sign of tissue pathology (pathoscore 3–4); moderate inflammation (pathoscore 5–8); and significant inflammation (pathoscore 9–13).

### Vaccination and challenge experiment

For vaccination study, three groups of wild type C57BL/6 mice (n = 10; each group) were pretreated with streptomycin according to the protocol described earlier [34]. Mice groups (3 groups; n = 5 mice each group) were vaccinated with MT5, MT4 strains and PBS respectively; the mice group treated with PBS served as a negative control group [34,48]. Fecal samples from each mice group were collected weekly and plated on MacConkey agar plate for analysis of fecal shedding of the vaccine strain. At day 30 post vaccination (p.v.), the histopathology of cecal mucosa and bacterial loads of different tissues of vaccinated mice (n = 5; each group) were analyzed. Further, the gut wash and serum samples of vaccinated mice were collected to assess serum IgG and gut secretory IgA (sIgA) by Western blot. The remaining mice (n = 5) from each vaccinated group were treated with ampicillin (25 mg by gavage) and challenged after 24 h with wild-type *S*. Typhimurium (SB300; 200 CFU) harboring ampicillin resistant plasmid pM973. The colonization efficiency of the challenged strain was evaluated at various host sites at day 3 post challenge (p.c.).

### Evaluation of serum and gut antibody response

To measure the mucosal immune response, serum IgG and secretory gut IgA responses were quantified by Western blot as described previously [34,48]. Serum and gut washes were collected at day 30 p.v from MT5 and MT4 immunized mice and the PBS treated control mice. The protein fractions of lysates from the overnight-grown *S.* Typhimurium wild-type strain (SB300), *ssaV* mutant (MT5), *ssaV* and *mig-14* double mutant (MT4) and *S.* Enteritidis P125109 (M1525) wild-type strain were separated on polyacrylamide gels and transferred to nitrocellulose membrane. The membrane was treated with suitably diluted serum sample or gut washes followed by incubation with conjugated α-mouse IgG (for serum; Santa cruz) and α-mouse IgA (for gut wash; Santa cruz). The blots were developed by ECL kit (Thermo Scientific).

### Statistical analysis

Statistical analyses were performed using the two-way ANOVA (GraphPad Prism 5). p < 0.05 was considered statistically significant.

## Results and discussion

### Additional *mig-14* mutation in *S.* Typhimurium *ssaV* mutant shows significant attenuation in immunocompromised mice

**Figure 1:**
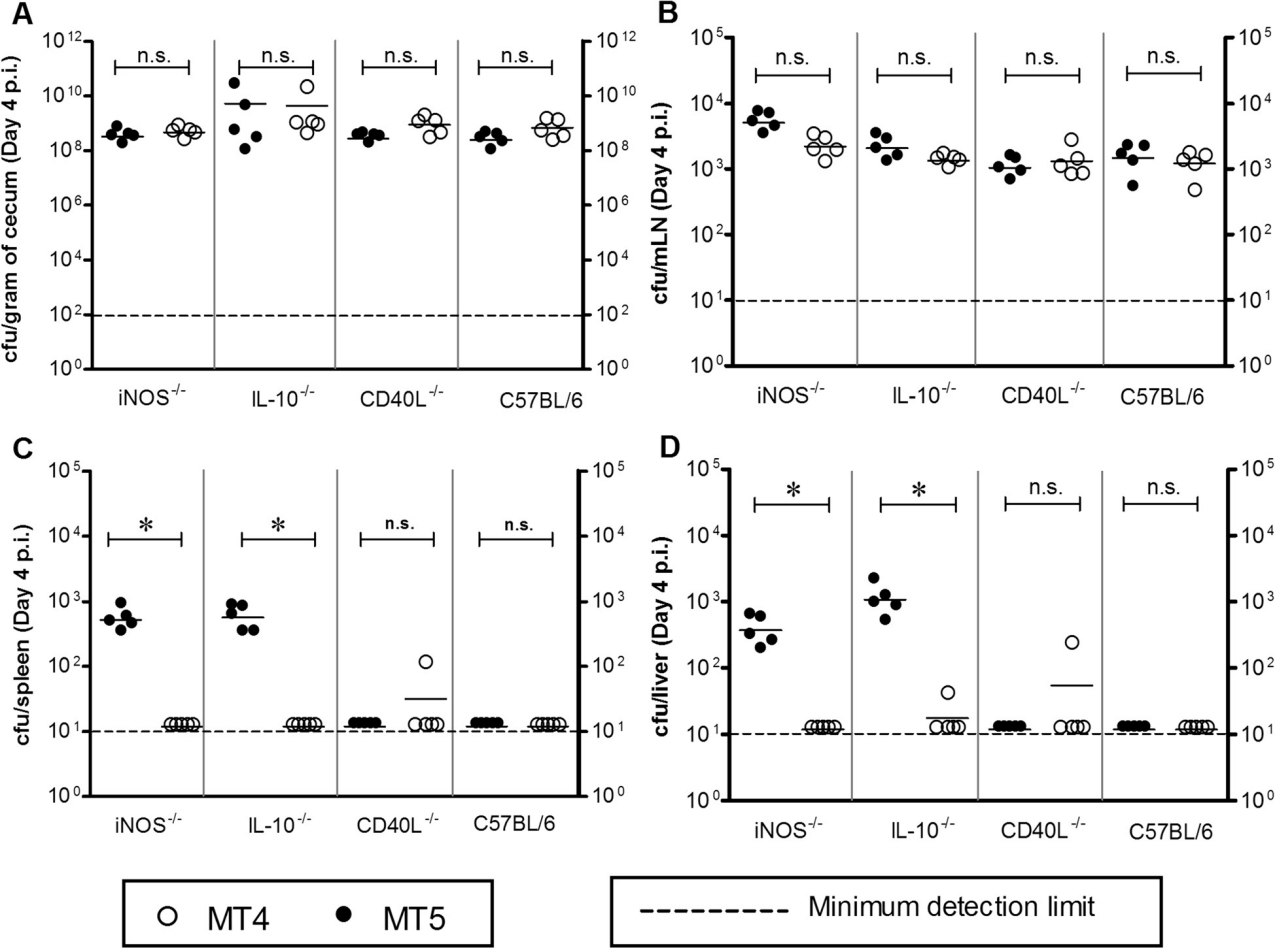
**Analysis of MT4 attenuation in comparison to MT5 in *Nos2***^**−/−**^**, *Il-10***^***−/−***^***, CD40L***^***−/−***^**and wild-type C57BL/6 mice.** Streptomycin pretreated mice were infected either with MT5 or MT4 (5x107 CFU by gavage; n = 5mice). Bacterial loads in cecum content **(A)**, mLN **(B)**, spleen **(C)** and liver **(D)** were assessed by plating at day 4 p.i.. n.s., statistically not significant; *, statistically significant (p < 0.05, Two-way ANOVA).

The attenuation of MT5 and MT4 strains in various immunocompromised mice was analyzed by normal infection experiment at day 4 p.i. In our initial observations, equivalent loads of MT5 and MT4 strains were detected in the cecal content of *Nos2*^−/−^, *Il-10*^*−/−*^ mice (Figure 1A) whereas, MT4 showed reduced colonization in spleen and liver (Figure 1B, C and D) as compared to MT5. Similar experiment was carried out to assess the performance of MT4 in wt C57BL/6 and *CD40L*^*−/−*^ mice. It was observed that neither MT4 nor MT5 colonized spleen and liver of *CD40L*^*−/−*^ and wild-type C57BL/6 mice (Figure 1C-D). However, MT4 (*ssaV*, *mig-14* mutant) colonized the mLN of wild-type mice as efficiently as MT5 (*ssaV* mutant) (Figure 1B). We also tested the attenuation profile in terms of competitive index of *mig14::aphT* single mutant against wild-type *S*. Typhimurium strain; it was appreciable that the *mig14::aphT* single mutant has reduced ability to colonize to systemic sites (Additional file 1: Figure S1 and Additional file 1: Figure S2); however, this reduced colonization in liver and spleen was not as sharp as in case of C57BL/6 mice infected with *ssaV* mutant MT5 (compare Additional file 1: Figure S2 with Figure 1C,D). Overall the data demonstrates that the deletion of *mig-14* in the *ssaV* knockout background does not allow *S*. Typhimurium to colonize the systemic sites like liver and spleen in severely immunocompromised mice (Figure 1C and D).

### MT4 protects wild-type C57BL/6 mice when challenged with wild-type *S*. Typhimurium

**Figure 2:**
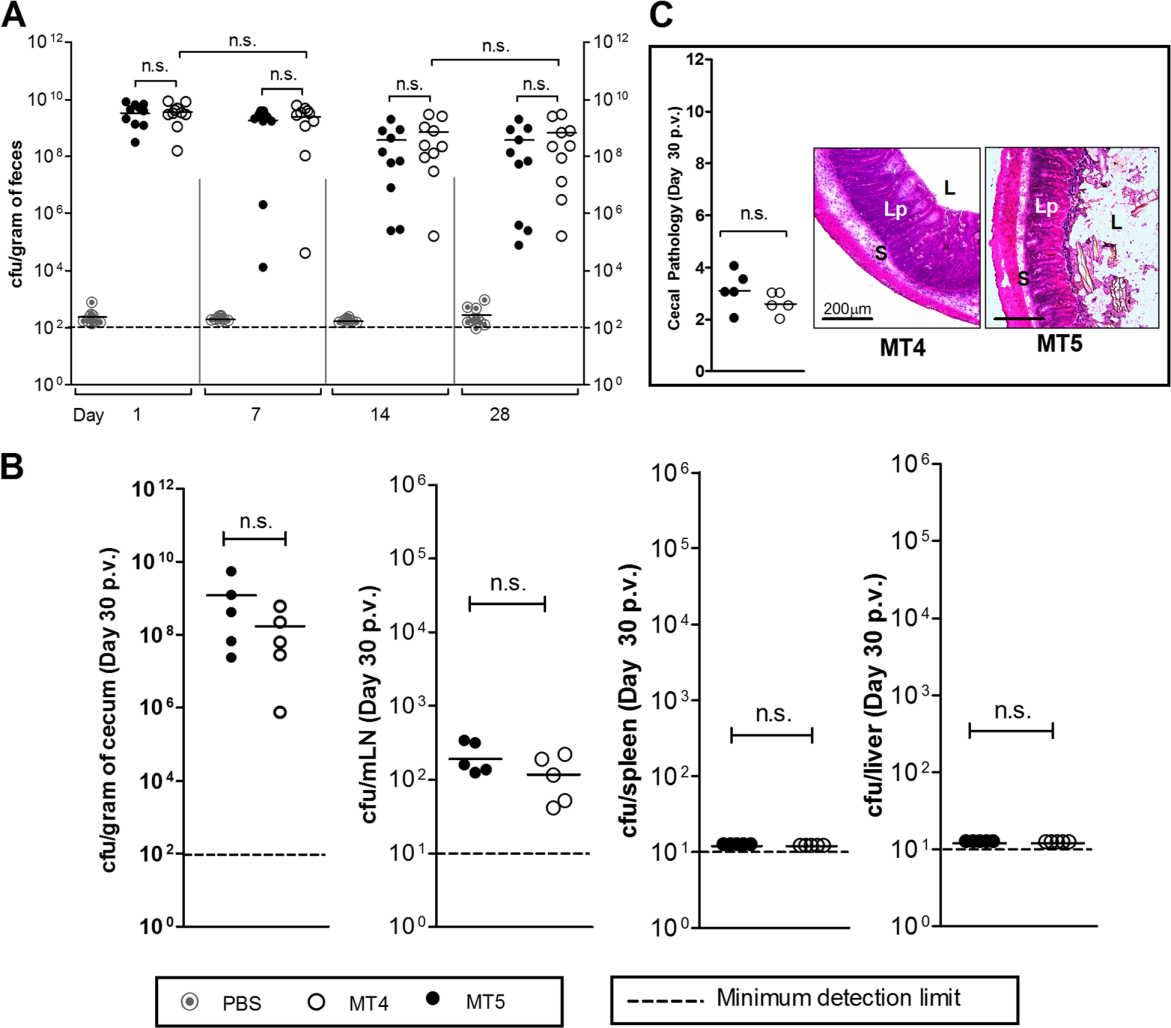
**Vaccination experiment analyzing the attenuation of MT4 at day 30 p.v.** For vaccination, C57BL/6 mice were treated with PBS (n = 10; grey solid circles), MT5 (5x10^7^CFU; n = 10; black solid circle) and MT4 (5x10^7^ CFU; n = 10; open circle). **(A)** Fecal shedding as analyzed by plating. PBS-controls: below detection limit (stripped line); **(B)** Colonization by the vaccine strains (MT5, n = 5 and MT4, n = 5) in cecal content, mLN, spleen and liver; **(C)** Cecal pathology at day 30 p.v.. n.s., not significant; *, statistically significant (p < 0.05).

**Figure 3:**
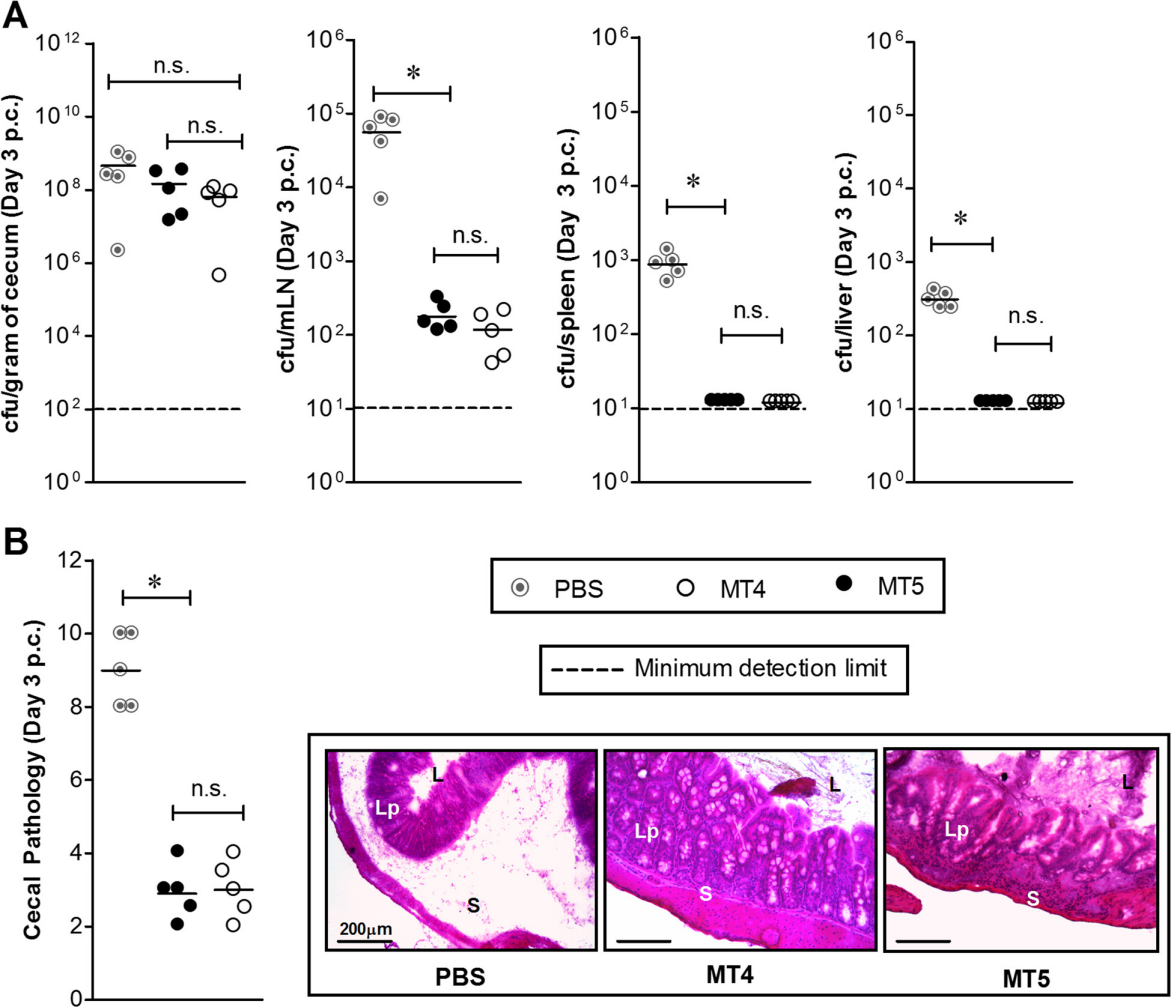
**Analysis of colonization and cecal pathology of the vaccinated mice after wild-type *S*. Typhimurium challenge.** Mice immunized with PBS, MT5 and MT4 (n = 5) were treated with ampicillin (25 mg by gavage), challenged with wild-type SB300 (amp^r^, sm^r^) and sacrificed three days later (day 3 p.c.). Disease parameters like colonization at various host-tissues **(A)** and cecal pathology **(B)** were determined. n.s., not significant; *, statistically significant (p < 0.05).

The immunogenic potential of MT4 in wild-type C57BL/6 mice was analyzed by previously established vaccination and challenge protocol using TTSS-2 deficient *S*. Typhimurium strain [34]. Three groups of wild-type C57BL/6 mice were vaccinated with MT4 (n = 10), MT5 (n = 10) and PBS (negative control; n = 10). The fecal shedding was analyzed as a measure of cecal colonization during vaccination period. Both, MT5 and MT4 strains reached a bacterial load of ~10^9^ CFU/g (of cecal content) in the gut lumen at the day 1 p.v.; however, the bacterial loads slightly declined at day 14 and day 28 p.v. (Figure 2A). Half the number of vaccinated mice (MT5, n = 5; MT4, n = 5; PBS, n = 5) were sacrificed to analyze cecal inflammation and the colonization levels in different systemic sites at day 30 p.i. With both the strains, cecum colonization was maintained up to ~10^7-9^ CFU/g. The bacterial load in mLN was lower as compared to the acute infection experiments (compare Figure 1B to 2B) whereas cecal mucosa did not show any sign of disease (Figure 2C). The remaining mice were analyzed for protection against a challenge with wild-type *S*. Typhimurium. At day 30 p.v., the remaining vaccinated mice (MT4, n = 5; MT5, n = 5; PBS, n = 5) were treated with 20 mg of ampicillin to remove regrown gut flora and any residual vaccine strain. Mice groups were then challenged with wild-type *S*. Typhimurium at day 31^st^ (200 CFU by gavage). The wild-type *S*. Typhimurium was able to colonize the lumen efficiently and reached the carrying capacity by day 3 p.c. in all three immunized groups (Figure 3A). Mice in the PBS treated control group suffered from severe enteropathy (Figure 3B). In contrast, the mice immunized with MT5 and MT4 strains did not show any signs of mucosal inflammation (Figure 3B). Furthermore, spleen and liver colonization by wild-type *S*. Typhimurium was significantly reduced in both the vaccinated groups (p < 0.05; Figure 3A). Thus, the data indicates that MT4 strain conferred equivalent level of protection from *Salmonella* inflicted disease as MT5 strain.

### Mice immunized with MT4 and MT5 showed equivalent response for both luminal IgA and serum specific IgG

**Figure 4:**
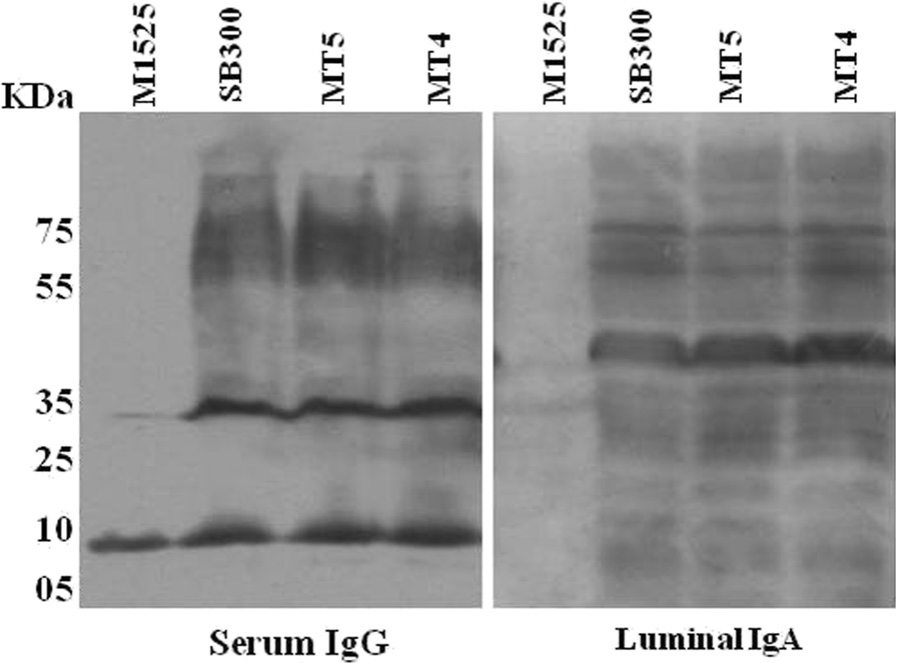
**Validation of antibody response (serum IgG and intestinal sIgA).** Serum and gut wash from mice treated with PBS and vaccinated with MT4 and MT5 were collected, diluted to a highest dilution of 1:120 (serum) and 1:9 (gut wash). The presence of *Salmonella* specific IgG and secretory IgA were detected by Western blots. The representative Western blot analysis of the antibody responses was done by developing the blots of overnight grown cultures of MT5, MT4, SB300 (wild-type *S*. Typhimurium) and M1525 (*S*. Enteritidis; negative control) with the serum and gut wash of the immunized mice.

Earlier it has been established that immune-protection against *S*. Typhimurium is based on O-antigen specific luminal sIgA along with serum IgA, IgM and IgG responses [34]. To validate the immunogenic potential of MT4, the antibody titers of IgG from serum and IgA from gut wash samples of mice vaccinated with MT4 and MT5 strains were detected by western blotting at the end of the day 30 p.v. (Figure 4). This experiment relies on the specific antibody binding to specific antigens of the bacterium (wild-type *S*. Typhimurium) as compared to a bacterium of different serovar (wild-type *S.* Enteritidis). The intestinal wash and serum samples from mice vaccinated with either MT5 or MT4 exhibited equivalent antibody response of *Salmonella* specific serum IgG and luminal secretory IgA. We additionally tested the antibody response through flow cytometry analysis and the data supported the finding that MT4 or MT5 vaccination exhibits equivalent antibody response (Additional file 1: Figure S4). The T-cytotoxic and T-helper cells play a critical role in the clearance of *Salmonella* as well as in the production of specific antibodies during the late phase of infection. We analyzed the effect of MT5 and MT4 strains on T-cell population of the mesenteric lymph node. We quantified the CD4^+^ and CD8^+^ T-cell population recovered from the mLN of the vaccinated mice after day 30 p.v. The T-cell population were analyzed by flowcytometry and found to be almost equally populated in the vaccinated mice but significantly more in comparison to the PBS treated mice (Additional file 1: Figure S3). This gives a sign that, the MT4 strain has an ability to colonize and induce T-cell mediated innate and adaptive immune response in the wild-type C57BL/6 mice.

## Conclusions

*S.* Typhimurium with a nonfunctional SPI-2 is considered as an avirulent and a potential vaccine strain [37]. In this study we have experimentally proved that *S*. Typhimurium diarrhea vaccine strain with nonfunctional SPI-2 system can be further attenuated without impeding the immunogenicity in immunocompromised hosts. We additionally mutated *mig-14* in *ssaV* deficient *S*. Typhimurium strain. The *ssaV*, *mig-14* double mutant was found to be highly attenuated in wild-type C57BL/6 mice and in immunocompromised mice like *Nos2*^*−/−*^*, Il-10*^*−/−*^ and *CD40L*^*−/−*^. These transgenic immunocompromised mice were selected for this study because of their high susceptibility to different infections [33,49,50]. One of the characteristic features of *Salmonella* infections in humans is that few infected individuals can become chronic carriers. Such individuals comprise about 1–6% of the total infected population [19,24] acting as reservoirs, and restricting the pathogen within the human populations. Previous studies have established that the successive progression of host-adapted *Salmonella* species has led to an increased virulence because of their association with the host along with increased invasiveness and long-term persistence [51,52]. The virulence factors essential for long-term persistence of the pathogen in their respective hosts are therefore likely to be important for its evolutionary success.

Mig-14 is an important factor for *Salmonella* resistance to IFN-γ-mediated host responses and to different anti-microbial peptide during the establishment of infection as well as survival in the macrophages [16]. It has also been reported that *mig-14* mutant can establish an infection but cannot persist for longer periods in the host system [53]. These reports support the contribution of Mig-14 in *Salmonella* long-term virulence. Although the mechanism of Mig-14 action is not completely established, the binding of Mig-14 deficient *Salmonella* to cathelin-related antimicrobial peptide (CRAMP) proves its active involvement in *Salmonella* antimicrobial peptide resistance [40]. Mechanistically, Mig-14 protein is a periplasmic protein which is tightly associated with the inner membrane of *Salmonella*[53]*.* The transmission electron microscopy study has revealed that the primary site of host CRAMP activity is the bacterial cytoplasm. Study of inner membrane localization of Mig-14 and cytoplasmic CRAMP activity, possibly suggests the role of Mig-14 in preventing penetration of CRAMP into the cytoplasm [40]. Taken together, these reports explain contribution of *mig-14* towards pathogen survival by encountering host inflammatory responses and promoting both acute and persistent bacterial infection. Therefore, in the present study, *mig-14* was taken as an important virulence factor to be knocked out from the existing live attenuated strain (MT5) with the goal to improve the attenuation attributes in immunocompromised mice.

In this study, we have assessed the degree of attenuation of *S*. Typhimurium *ssaV* mutant (MT5) and *ssaV*, *mig-*14 double mutant (MT4) in immunocompromised mice, by infecting these two strains to *Nos2*^*−/−*^*, Il-10*^*−/−*^*and CD40L*^*−/−*^ C57BL/6 mice. The day 4 p.i. observation showed a high degree of systemic attenuation of MT4 (*ssaV*, *mig-14*) strain in *Nos2*^*−/−*^*, Il-10*^*−/−*^ mice in comparison to the MT5 (*ssaV*) strain. On the other hand MT5 and MT4 strains were equally attenuated in *CD40L*^*−/−*^ mice. Interestingly, MT4 strain also retained its capacity to colonize the mesenteric lymph node of *Nos2*^*−/−*^*, Il-10*^*−/−*^*and CD40L*^*−/−*^ mice, demonstrating its ability to access the mLN but not the systemic sites. The *in vivo* data showed that the attenuation of MT4 in immunocompromised mice could be due to the absence of *mig-14* in *ssaV* deficient *S*. Typhimurium. Furthermore, the MT4 and MT5 strains were used to vaccinate the wild-type C57BL/6 mice. Results showed that none of the mice developed cecal inflammation at day 30 p.v. However, both the strains (MT5 and MT4) equally colonized the gut lumen of vaccinated mice groups. Apart from this, at 30 day p. v., neither of the strain was found in the systemic organs which diminishes the possibility of late systemic dissemination and associated disease symptoms. Interestingly, apart from MT5, we also found a small population of MT4 strain in the mesenteric lymph node of the immunized mice, showing the potential of MT4 to stay in the lymphoid tissue for a longer period. In a challenge experiment, the vaccinated mice were protected when challenged with wild-type *S*. Typhimurium, however, the PBS treated mice developed significant inflammation and systemic dissemination of *S*. Typhimurium during subsequent *Salmonella* challenge.

In conclusion, the MT4 live-attenuated *S*. Typhimurium strain provides an efficient antibody mediated immune response which can protect even immunocompromised hosts from lethal infection of *Salmonella.* Specific antibody response to any protein antigens requires the involvement of both CD4^+^ and CD8^+^ T-cells along with the B-cells. The T-cell dependent antigens require the involvement of T-cells for the adaptive immune response. T helper (CD4^+^) cells play a vital role in stimulating the B-cells for the production of pathogen specific antibody via clonal propagation. Additionally, the activated CD4^+^ and CD8^+^ T-cells are the major producers of INF-γ which further activates the tissue and blood macrophages. As T-cell contributes to the cell mediated immune response, it is important to estimate the T-cell propagation during the course of *Salmonella* infection. In this study we have additionally estimated CD4^+^ and CD8^+^ T-cells from the mLN of the immunized mice. CD4^+^ and CD8^+^ T-cell population of the mice immunized with MT4 strain found to be comparable with the mice immunized with MT5 strain. Hence, it concludes that the MT4 strain retains its ability to induce the classical innate and adaptive immune response even after a strong attenuation. Therefore, we propose that incorporating additional “safety” features such as the deletion of *mig-14* can be of a general interest for the design of new super live attenuated *S*. Typhimurium strain. This attenuated strain could also be used for developing the recombinant vaccine against other enteric pathogens.

## Competing interests

The authors declare that they have no competing interests.

## Authors’ contributions

NB, BS, MS designed the experiments; NP, VV, SS performed the experiments; NB, NS, MS wrote the manuscript; VV, NS, SD, MS reviewed and edited the manuscript; BS provided the animal facility; MS provided the chemicals and consumables for this study. All authors read and approved the final manuscript.

## Supplementary Material

Additional file 1: Figure S1 Evaluation of attenuation profile of *mig14::aphT* mutant in comparison to wild-type strain of *Salmonella* Typhimurium. Competitive index profile of mig-14::aphT mutant when compared against wild-type strain. n.s. = not significant; * = p < 0.05). **Figure S2.** Infection profile of mig14::aphT mutant in comparison to wild-type strain of Salmonella Typhimurium .Infection profile and systemic attenuation of *mig14::aphT* mutant. Bar indicates 200 μm. n.s. = not significant; * = p < 0.05). **Figure S3.** Flowcytometric analysis of T-cell population after *Salmonella* infection. The whole cells were isolated from the mLN of the vaccinated mice. The cells were then suspended in appropriate medium and processed for flow cytometric analysis (see materials and methods). The cells were detected by using specific conjugated antibodies against specific T-cells. **Figure S4.** Luminal and serum specific antibody responses in mice immunized with MT5 and MT4. Serum and gut wash from mice treated with PBS and vaccinated with MT4 and MT5 were collected, diluted to a highest dilution of 1:120 (serum) and 1:9 (gut wash). The presence of *Salmonella* specific IgG and secretory IgA were detected by bacterial flow cytometric (A) and Western blot (B). Each coloured line indicates data obtained from individual mice of respective group. The representative Western blot analysis of the antibody responses was done by developing the blots from the overnight cultures of MT5, MT4, SB300 (wt *S*. Typhimurium) and M1525 (*S*. Enteritidis; negative control) by using the sera and gut luminal sIgA of the immunized mice. Click here for file

## References

[B1] Okamura M Lillehoj HS Raybourne RB Babu US Heckert RA Cell-mediated immune responses to a killed Salmonella enteritidis vaccine: lymphocyte proliferation, T-cell changes and interleukin-6 (IL-6), IL-1, IL-2, and IFN-gamma production Comp Immunol Microbiol Infect Dis 2004 27 4 255 272 10.1016/j.cimid.2003.12.001 15178000

[B2] Thatte J Rath S Bal V Analysis of immunization route-related variation in the immune response to heat-killed Salmonella typhimurium in mice Infect Immun 1995 63 1 99 103 7806391 10.1128/iai.63.1.99-103.1995 PMC172963

[B3] Penha Filho RA Moura BS de Almeida AM Montassier HJ Barrow PA Berchieri Junior A Humoral and cellular immune response generated by different vaccine programs before and after Salmonella Enteritidis challenge in chickens Vaccine 2012 30 52 7637 7643 10.1016/j.vaccine.2012.10.020 23085366

[B4] Crhanova M Hradecka H Faldynova M Matulova M Havlickova H Sisak F Rychlik I Immune response of chicken gut to natural colonization by gut microflora and to Salmonella enterica serovar enteritidis infection Infect Immun 2011 79 7 2755 2763 10.1128/IAI.01375-10 21555397 PMC3191970

[B5] Silva EN Snoeyenbos GH Weinack OM Smyser CF Studies on the use of 9R strain of Salmonella gallinarum as a vaccine in chickens Avian Dis 1981 25 1 38 52 10.2307/1589825 7271663

[B6] Roland K Tinge S Warner E Sizemore D Comparison of different attenuation strategies in development of a Salmonella hadar vaccine Avian Dis 2004 48 3 445 452 10.1637/7106 15529966

[B7] Robertsson JA Lindberg AA Hoiseth S Stocker BA Salmonella typhimurium infection in calves: protection and survival of virulent challenge bacteria after immunization with live or inactivated vaccines Infect Immun 1983 41 2 742 750 6347895 10.1128/iai.41.2.742-750.1983 PMC264704

[B8] Vladoianu IR Dubini F Experimental model of oral antityphoid vaccination with live streptomycin-dependent Salmonella typhimurium in C57BL/6 mice J Hyg (Lond) 1975 75 2 215 218 10.1017/S0022172400047240 1100712 PMC2130300

[B9] Totemeyer S Kaiser P Maskell DJ Bryant CE Sublethal infection of C57BL/6 mice with Salmonella enterica Serovar Typhimurium leads to an increase in levels of Toll-like receptor 1 (TLR1), TLR2, and TLR9 mRNA as well as a decrease in levels of TLR6 mRNA in infected organs Infect Immun 2005 73 3 1873 1878 10.1128/IAI.73.3.1873-1878.2005 15731092 PMC1064909

[B10] Vishwakarma V Pati NB Chandel HS Sahoo SS Saha B Suar M Evaluation of Salmonella enterica serovar Typhimurium TTSS-2 deficient fur mutant as safe live-attenuated vaccine candidate for immunocompromised mice PLoS One 2012 7 12 e52043 10.1371/journal.pone.0052043 23284865 PMC3524104

[B11] Toobak H Rasooli I Talei D Jahangiri A Owlia P Darvish Alipour Astaneh S Immune response variations to Salmonella enterica serovar Typhi recombinant porin proteins in mice Biologicals 2013 41 4 224 230 10.1016/j.biologicals.2013.05.005 23796754

[B12] Chaudhuri RR Peters SE Pleasance SJ Northen H Willers C Paterson GK Cone DB Allen AG Owen PJ Shalom G Comprehensive identification of Salmonella enterica serovar typhimurium genes required for infection of BALB/c mice PLoS Pathog 2009 5 7 e1000529 10.1371/journal.ppat.1000529 19649318 PMC2712085

[B13] Cheminay C Hensel M Rational design of Salmonella recombinant vaccines Int J Med Microbiol 2008 298 1–2 87 98 17888730 10.1016/j.ijmm.2007.08.006

[B14] Gilks CF Brindle RJ Otieno LS Simani PM Newnham RS Bhatt SM Lule GN Okelo GB Watkins WM Waiyaki PG Life-threatening bacteraemia in HIV-1 seropositive adults admitted to hospital in Nairobi, Kenya Lancet 1990 336 8714 545 549 10.1016/0140-6736(90)92096-Z 1975046

[B15] Gordon MA Banda HT Gondwe M Gordon SB Boeree MJ Walsh AL Corkill JE Hart CA Gilks CF Molyneux ME Non-typhoidal salmonella bacteraemia among HIV-infected Malawian adults: high mortality and frequent recrudescence Aids 2002 16 12 1633 1641 10.1097/00002030-200208160-00009 12172085

[B16] Raupach B Kaufmann SH Bacterial virulence, proinflammatory cytokines and host immunity: how to choose the appropriate Salmonella vaccine strain? Microbes Infect 2001 3 14–15 1261 1269 11755414 10.1016/s1286-4579(01)01486-1

[B17] Dunstan SJ Simmons CP Strugnell RA Comparison of the abilities of different attenuated Salmonella typhimurium strains to elicit humoral immune responses against a heterologous antigen Infect Immun 1998 66 2 732 740 9453634 10.1128/iai.66.2.732-740.1998 PMC107964

[B18] Garmory HS Leary SE Griffin KF Williamson ED Brown KA Titball RW The use of live attenuated bacteria as a delivery system for heterologous antigens J Drug Target 2003 11 8–10 471 479 15203915 10.1080/10611860410001670008

[B19] Hohmann EL Oletta CA Miller SI Evaluation of a phoP/phoQ-deleted, aroA-deleted live oral Salmonella typhi vaccine strain in human volunteers Vaccine 1996 14 1 19 24 10.1016/0264-410X(95)00173-X 8821644

[B20] Tacket CO Kelly SM Schodel F Losonsky G Nataro JP Edelman R Levine MM Curtiss R 3rd Safety and immunogenicity in humans of an attenuated Salmonella typhi vaccine vector strain expressing plasmid-encoded hepatitis B antigens stabilized by the Asd-balanced lethal vector system Infect Immun 1997 65 8 3381 3385 9234801 10.1128/iai.65.8.3381-3385.1997 PMC175478

[B21] Chatfield SN Strugnell RA Dougan G Live Salmonella as vaccines and carriers of foreign antigenic determinants Vaccine 1989 7 6 495 498 10.1016/0264-410X(89)90271-5 2481908

[B22] Curtiss R 3rd Wanda SY Gunn BM Zhang X Tinge SA Ananthnarayan V Mo H Wang S Kong W Salmonella enterica serovar typhimurium strains with regulated delayed attenuation in vivo Infect Immun 2009 77 3 1071 1082 10.1128/IAI.00693-08 19103774 PMC2643627

[B23] Heithoff DM Enioutina EY Daynes RA Sinsheimer RL Low DA Mahan MJ Salmonella DNA adenine methylase mutants confer cross-protective immunity Infect Immun 2001 69 11 6725 6730 10.1128/IAI.69.11.6725-6730.2001 11598044 PMC100049

[B24] Matsui H Suzuki M Isshiki Y Kodama C Eguchi M Kikuchi Y Motokawa K Takaya A Tomoyasu T Yamamoto T Oral immunization with ATP-dependent protease-deficient mutants protects mice against subsequent oral challenge with virulent Salmonella enterica serovar typhimurium Infect Immun 2003 71 1 30 39 10.1128/IAI.71.1.30-39.2003 12496146 PMC143154

[B25] McFarland WC Stocker BA Effect of different purine auxotrophic mutations on mouse-virulence of a Vi-positive strain of Salmonella dublin and of two strains of Salmonella typhimurium Microb Pathog 1987 3 2 129 141 10.1016/0882-4010(87)90071-4 2849016

[B26] Miller SI Loomis WP Alpuche-Aranda C Behlau I Hohmann E The PhoP virulence regulon and live oral Salmonella vaccines Vaccine 1993 11 2 122 125 10.1016/0264-410X(93)90006-J 8438611

[B27] Angelakopoulos H Hohmann EL Pilot study of phoP/phoQ-deleted Salmonella enterica serovar typhimurium expressing Helicobacter pylori urease in adult volunteers Infect Immun 2000 68 4 2135 2141 10.1128/IAI.68.4.2135-2141.2000 10722611 PMC97395

[B28] Hindle Z Chatfield SN Phillimore J Bentley M Johnson J Cosgrove CA Ghaem-Maghami M Sexton A Khan M Brennan FR Characterization of Salmonella enterica derivatives harboring defined aroC and Salmonella pathogenicity island 2 type III secretion system (ssaV) mutations by immunization of healthy volunteers Infect Immun 2002 70 7 3457 3467 10.1128/IAI.70.7.3457-3467.2002 12065485 PMC128087

[B29] Toso JF Gill VJ Hwu P Marincola FM Restifo NP Schwartzentruber DJ Sherry RM Topalian SL Yang JC Stock F Phase I study of the intravenous administration of attenuated Salmonella typhimurium to patients with metastatic melanoma J Clin Oncol 2002 20 1 142 152 10.1200/JCO.20.1.142 11773163 PMC2064865

[B30] Hone DM Tacket CO Harris AM Kay B Losonsky G Levine MM Evaluation in volunteers of a candidate live oral attenuated Salmonella typhi vector vaccine J Clin Invest 1992 90 2 412 420 10.1172/JCI115876 1644914 PMC443116

[B31] Dilts DA Riesenfeld-Orn I Fulginiti JP Ekwall E Granert C Nonenmacher J Brey RN Cryz SJ Karlsson K Bergman K Phase I clinical trials of aroA aroD and aroA aroD htrA attenuated S. typhi vaccines; effect of formulation on safety and immunogenicity Vaccine 2000 18 15 1473 1484 10.1016/S0264-410X(99)00424-7 10618545

[B32] Kotton CN Lankowski AJ Scott N Sisul D Chen LM Raschke K Borders G Boaz M Spentzou A Galan JE Safety and immunogenicity of attenuated Salmonella enterica serovar Typhimurium delivering an HIV-1 Gag antigen via the Salmonella Type III secretion system Vaccine 2006 24 37–39 6216 6224 16824652 10.1016/j.vaccine.2006.05.094

[B33] Kwon YM Cox MM Calhoun LN Salmonella-based vaccines for infectious diseases Expert Rev Vaccines 2007 6 2 147 152 10.1586/14760584.6.2.147 17408365

[B34] Endt K Stecher B Chaffron S Slack E Tchitchek N Benecke A Van Maele L Sirard JC Mueller AJ Heikenwalder M The microbiota mediates pathogen clearance from the gut lumen after non-typhoidal Salmonella diarrhea PLoS Pathog 2010 6 9 e1001097 10.1371/journal.ppat.1001097 20844578 PMC2936549

[B35] Hensel M Shea JE Gleeson C Jones MD Dalton E Holden DW Simultaneous identification of bacterial virulence genes by negative selection Science 1995 269 5222 400 403 10.1126/science.7618105 7618105

[B36] Shea JE Beuzon CR Gleeson C Mundy R Holden DW Influence of the Salmonella typhimurium pathogenicity island 2 type III secretion system on bacterial growth in the mouse Infect Immun 1999 67 1 213 219 9864218 10.1128/iai.67.1.213-219.1999 PMC96299

[B37] Periaswamy B Maier L Vishwakarma V Slack E Kremer M Andrews-Polymenis HL McClelland M Grant AJ Suar M Hardt WD Live attenuated S. Typhimurium vaccine with improved safety in immuno-compromised mice PLoS One 2012 7 9 e45433 10.1371/journal.pone.0045433 23029007 PMC3454430

[B38] Fang FC Antimicrobial reactive oxygen and nitrogen species: concepts and controversies Nat Rev Microbiol 2004 2 10 820 832 10.1038/nrmicro1004 15378046

[B39] Valdivia RH Cirillo DM Lee AK Bouley DM Falkow S mig-14 is a horizontally acquired, host-induced gene required for salmonella enterica lethal infection in the murine model of typhoid fever Infect Immun 2000 68 12 7126 7131 10.1128/IAI.68.12.7126-7131.2000 11083839 PMC97824

[B40] Brodsky IE Ghori N Falkow S Monack D Mig-14 is an inner membrane-associated protein that promotes Salmonella typhimurium resistance to CRAMP, survival within activated macrophages and persistent infection Mol Microbiol 2005 55 3 954 972 15661016 10.1111/j.1365-2958.2004.04444.x

[B41] Hoiseth SK Stocker BA Aromatic-dependent Salmonella typhimurium are non-virulent and effective as live vaccines Nature 1981 291 5812 238 239 10.1038/291238a0 7015147

[B42] Vishwakarma V Periaswamy B Bhusan Pati N Slack E Hardt WD Suar M A novel phage element of Salmonella enterica serovar Enteritidis P125109 contributes to accelerated type III secretion system 2-dependent early inflammation kinetics in a mouse colitis model Infect Immun 2012 80 9 3236 3246 10.1128/IAI.00180-12 22753379 PMC3418750

[B43] Datsenko KA Wanner BL One-step inactivation of chromosomal genes in Escherichia coli K-12 using PCR products Proc Natl Acad Sci USA 2000 97 12 6640 6645 10.1073/pnas.120163297 10829079 PMC18686

[B44] Hapfelmeier S Stecher B Barthel M Kremer M Muller AJ Heikenwalder M Stallmach T Hensel M Pfeffer K Akira S The Salmonella pathogenicity island (SPI)-2 and SPI-1 type III secretion systems allow Salmonella serovar typhimurium to trigger colitis via MyD88-dependent and MyD88-independent mechanisms J Immunol 2005 174 3 1675 1685 15661931 10.4049/jimmunol.174.3.1675

[B45] Barthel M Hapfelmeier S Quintanilla-Martinez L Kremer M Rohde M Hogardt M Pfeffer K Russmann H Hardt WD Pretreatment of mice with streptomycin provides a Salmonella enterica serovar Typhimurium colitis model that allows analysis of both pathogen and host Infect Immun 2003 71 5 2839 2858 10.1128/IAI.71.5.2839-2858.2003 12704158 PMC153285

[B46] Suar M Jantsch J Hapfelmeier S Kremer M Stallmach T Barrow PA Hardt WD Virulence of broad- and narrow-host-range Salmonella enterica serovars in the streptomycin-pretreated mouse model Infect Immun 2006 74 1 632 644 10.1128/IAI.74.1.632-644.2006 16369020 PMC1346614

[B47] Suar M Periaswamy B Songhet P Misselwitz B Muller A Kappeli R Kremer M Heikenwalder M Hardt WD Accelerated type III secretion system 2-dependent enteropathogenesis by a Salmonella enterica serovar enteritidis PT4/6 strain Infect Immun 2009 77 9 3569 3577 10.1128/IAI.00511-09 19528213 PMC2738034

[B48] Endt K Maier L Kappeli R Barthel M Misselwitz B Kremer M Hardt WD Peroral ciprofloxacin therapy impairs the generation of a protective immune response in a mouse model for Salmonella enterica serovar Typhimurium diarrhea, while parenteral ceftriaxone therapy does not Antimicrob Agents Chemother 2012 56 5 2295 2304 10.1128/AAC.05819-11 22354292 PMC3346637

[B49] Andrews FJ Katz F Jones A Smith S Finn A CD40 ligand deficiency presenting as unresponsive neutropenia Arch Dis Child 1996 74 5 458 459 10.1136/adc.74.5.458 8669967 PMC1511550

[B50] Padigel UM Alexander J Farrell JP The role of interleukin-10 in susceptibility of BALB/c mice to infection with Leishmania mexicana and Leishmania amazonensis J Immunol 2003 171 7 3705 3710 14500669 10.4049/jimmunol.171.7.3705

[B51] Levine MM Black RE Lanata C Precise estimation of the numbers of chronic carriers of Salmonella typhi in Santiago, Chile, an endemic area J Infect Dis 1982 146 6 724 726 10.1093/infdis/146.6.724 7142746

[B52] Hoffman TA Ruiz CJ Counts GW Sachs JM Nitzkin JL Waterborne typhoid fever in Dade County, Florida. Clinical and therapeutic evaluation of 105 bacteremic patients Am J Med 1975 59 4 481 487 10.1016/0002-9343(75)90255-7 1166856

[B53] Brodsky IE Ernst RK Miller SI Falkow S mig-14 is a Salmonella gene that plays a role in bacterial resistance to antimicrobial peptides J Bacteriol 2002 184 12 3203 3213 10.1128/JB.184.12.3203-3213.2002 12029036 PMC135090

