# Supplementary material for: Salmonella Typhimurium TTSS-2 deficient mig-14 mutant shows attenuation in immunocompromised mice and offers protection against wild-type Salmonella Typhimurium infection
Source: BMC Microbiol. 2013 Oct 22;13:236. doi: 10.1186/1471-2180-13-236 (PMC3819739; doi:10.1186/1471-2180-13-236)
Supplement: Additional file 1: Figure S1 — Evaluation of attenuation profile of mig14::aphT mutant in comparison to wild-type strain of Salmonella Typhimurium. Competitive index profile of mig-14::aphT mutant when compared against wild-type strain. n.s. = not significant; * = p < 0.05). Figure S2. Infection profile of mig14::aphT mutant in comparison to wild-type strain of Salmonella Typhimurium .Infection profile and systemic attenuation of mig14::aphT mutant. Bar indicates 200 μm. n.s. = not significant; * = p < 0.05). Figure S3. Flowcytometric analysis of T-cell population after Salmonella infection. The whole cells were isolated from the mLN of the vaccinated mice. The cells were then suspended in appropriate medium and processed for flow cytometric analysis (see materials and methods). The cells were detected by using specific conjugated antibodies against specific T-cells. Figure S4. Luminal and serum specific antibody responses in mice immunized with MT5 and MT4. Serum and gut wash from mice treated with PBS and vaccinated with MT4 and MT5 were collected, diluted to a highest dilution of 1:120 (serum) and 1:9 (gut wash). The presence of Salmonella specific IgG and secretory IgA were detected by bacterial flow cytometric (A) and Western blot (B). Each coloured line indicates data obtained from individual mice of respective group. The representative Western blot analysis of the antibody responses was done by developing the blots from the overnight cultures of MT5, MT4, SB300 (wt S. Typhimurium) and M1525 (S. Enteritidis; negative control) by using the sera and gut luminal sIgA of the immunized mice. [file 1471-2180-13-236-S1.pdf]

**Supplementary figures:**

**S1- Evaluation of attenuation profile of *mig14::aphT* mutant in comparison to wild-type strain of *Salmonella* Typhimurium .**

**Methodology:** The group of streptomycin pretreated mice (n=5) was infected with mixed inoculum of mig14::aphT mutant and wild-type *Salmonella* Typhimurium strain (cfu $10^5$ each strain). Mice were kept in IVC under observation for 4 days post infection. Host tissues were excised, homogenised and pleated on streptomycin (Sm; for collective cfu count) and streptomycin-kanamycin (Sm-Km; for mig14::aphT mutant cfu count) supplemented Mac Conkey agar plates. The cfu count for wild-type strain was obtained by subtracting mig14::aphT counts from the collective counts obtained from respective tissue. Obtained individual counts were compared to get competitive index of mig14::aphT single mutant in comparison to wild-type *Salmonella* Typhimurium strain.

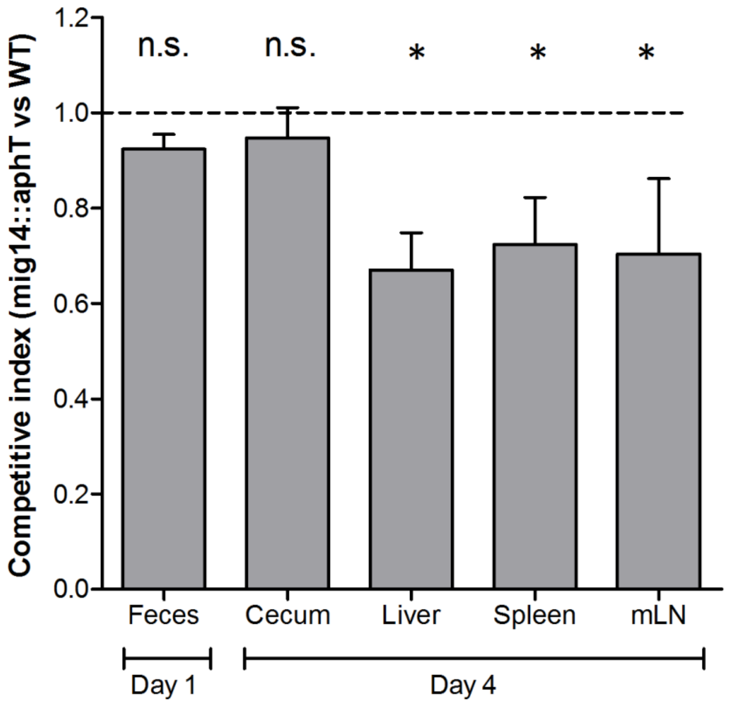


Fig. S1 : Competitive index profile of mig-14::aphT mutant when compared against wild-type strain. n.s.= not significant; ∗=P<0.05)

**Analysis:** We tested the competitive index of mig14 mutant in a group of five C57BL/6 mice. Bacterial strains were grown separately and mixed at the time of infection to prepare an inoculum mixture. A significant decrease (P<0.05) in the CI values for mLN, Liver and Spleen colonization was observed for *mig14::aphT* mutant when compared against wild-type *S.* Typhimurium strain. This result could possibly explain for the further attenuation of the developed double mutant (*ssaV*, *mig14*) when subjected to immunocompromised mice.

**S2- Infection profile of *mig14::aphT* mutant in comparison to wild-type strain of *Salmonella* Typhimurium .**

**Methodology:** Groups of streptomycin pretreated mice (n=5) were infected with wild-type strain, mig14::aphT mutant strain and mig14-complemented single mutant strain (cfu $10^5$ each strain). {The mig14 wild-type allele was cloned into NcoI-XbaI sites of plasmid pCH112 having pBAD backbone and arabinose inducible promoter with cloned segment of *hilA* gene. In this plasmid, the *hilA* gene was replaced with *mig-14* allele}. Mice were kept in IVC under observation for 4 days post infection. Host tissues were excised, homogenised and pleated on agr plates supplemented with suitable antibiotics. The cecal sections from each mice group were HE stained and analyzed.

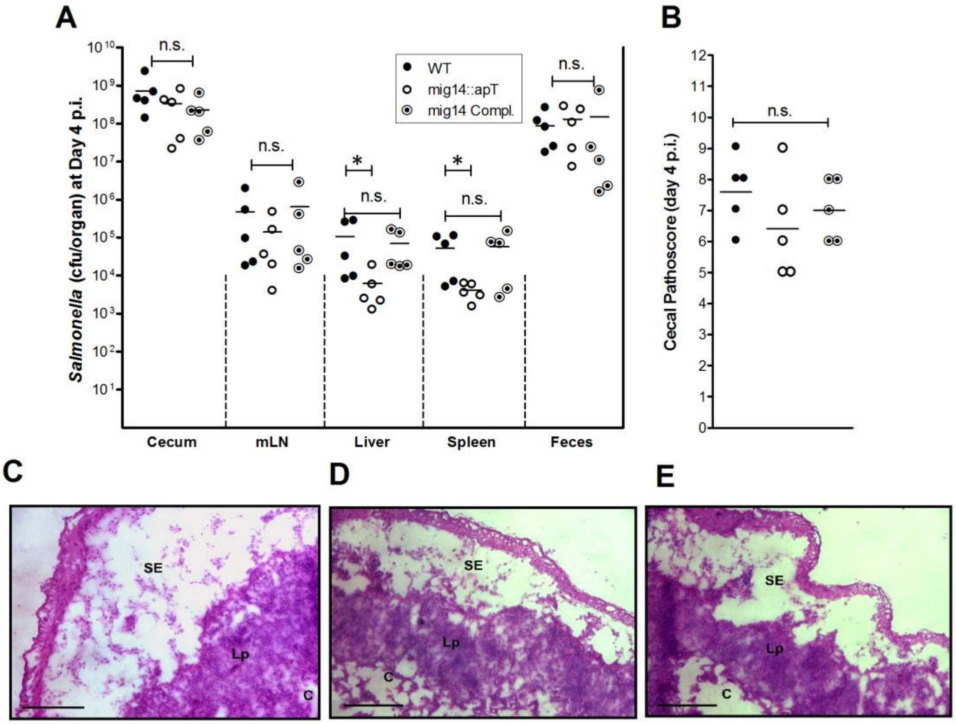


Fig. S2 : Infection profile and systemic attenuation of *mig14::aphT* mutant. Bar indicates 200µm. n.s.= not significant; ∗=P<0.05)

**Analysis:** The *mig14::aphT* mutant was compared against the wild-type SB300 strain (WT) for its systemic colonization and ability to cause cecal inflammation. Groups of streptomycin pretreated C57BL/6 mice (n=5 each) were infected with $10^5$ cfu of wild-type *S*.Typhimurium WT, *mig14::aphT* and *mig14*-complemented strain. The bacterial densities in fecal shedding (day 1 post infection) and the organ loads (day 4 p.i.) were assessed.

We observed no significant changes in the ability of *mig14::aphT* mutant strain in terms of cecal colonization when compared with wild-type *Salmonella* typhimurium strain. However, a significant reduction in the systemic infiltration was noticed in the mice infected with *mig14::aphT* mutant as compared to the WT infected mice group. This reduction was restored to much extent on complementing wild-type allele of mig14 to its respective mutant (*mig14::aphT*). The decrease in the ability of mig14::aphT mutant to colonize systemically was not that sharp as of *ssaV* mutant MT5 (Fig.1) but was noticeable, this provided enough hint for adding an additional mutation of *mig14* on *ssaV* mutant to possibly attain complete attenuation in immunocompromised mice group.

**S3- Flowcytometric analysis of T-cell population after *Salmonella* infection.**

**Methodology:** Analysis of the T-cell population was performed by collecting the mesenteric lymph node (mLN) of the vaccinated mice in 500 µl of *Rose Park Memorial Institut*e (RPMI) medium with 10% fetal calf serum (FCS). Collected mLN were homogenized and centrifuged at 1200 rpm. The pellet was suspended in 1ml RPMI with 10% FCS. About $10^6$ cells were resuspended in 200 µl FCS and incubated for 1 h in ice. 1ml of FACS buffer (1xPBS with 2% FCS or BSA) was added just after the incubation and spun at 12000 rpm for 8 min at $4^oC$.The cells were then resuspended with 50 µl of FACS buffer and incubated with T-cell specific antibodies (PE- mouse anti CD3, BD; PB- mouse anti CD4, BD; FITC- mouse anti CD8, BD) for 1 h and 30 min. The cells were subsequently washed for two times in the FACS buffer and fixed with 1% PFA (1 ml) for 15 min at $4^o$ C. Finally the cells were washed two times with the FACS buffer. The cell surface staining was quantified by FACS and analyzed by using BD FACSDiva software.

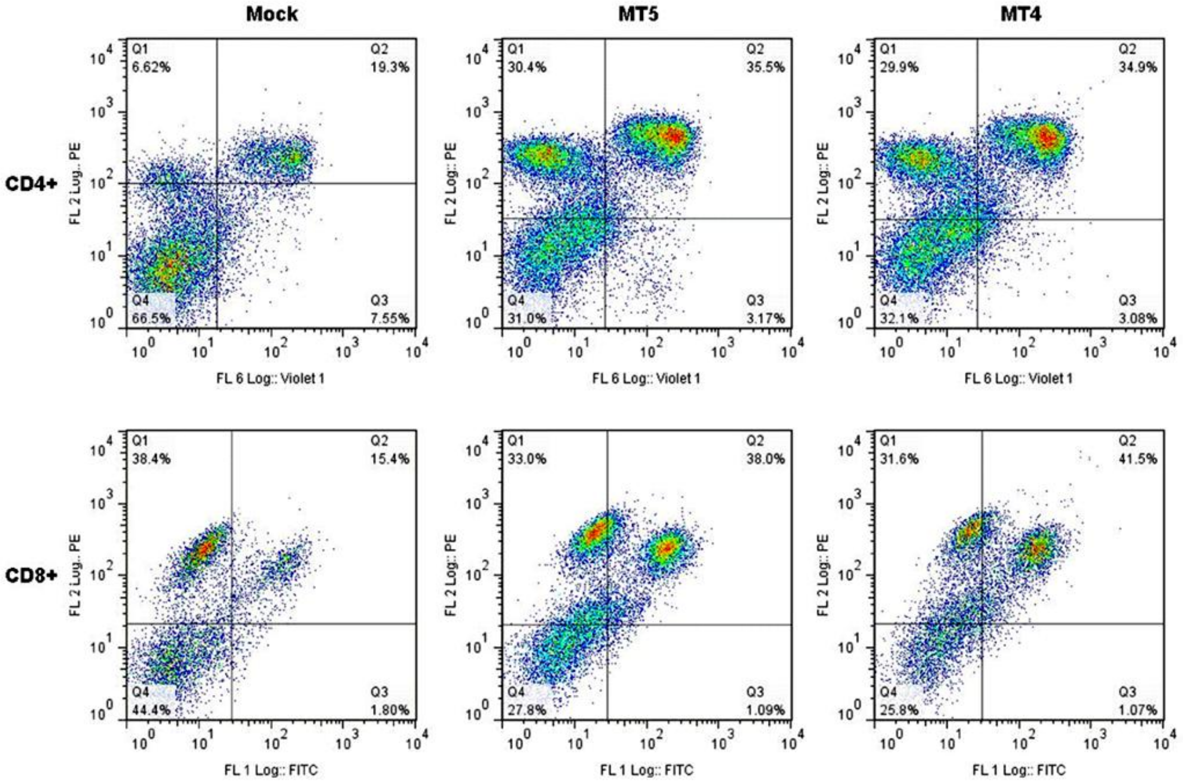


Fig. S3 : The whole cells were isolated from the mLN of the vaccinated mice. The cells were then suspended in appropriate medium and processed for FACS analysis (see materials and methods). The cells were detected by using specific conjugated antibodies against specific T-cells.

**Analysis:** An ideal live-attenuated vaccine strain should develop enough immunogenicity on reaching the host lymphoid tissues. The T-cytotoxic and T-helper cells play a critical role in the clearance of *Salmonella* as well as in the production of specific antibodies during the late phase of infection. We analyzed the effect of MT5 and MT4 strains on T-cell population of the mesenteric lymph node. We quantified the $CD4^+$ and $CD8^+$ T-cell population recovered from the mLN of the vaccinated mice after day 30 p.v. The T-cell population were analyzed by flowcytometry and found to be almost equally populated in the vaccinated mice but significantly more in comparison to the PBS treated mice. This clearly indicates that, the MT4 strain has an ability to colonize and induce T-cell mediated innate and adaptive immune response in the wild-type C57BL/6 mice.

## S4- Luminal and serum specific antibody responses in mice immunized with MT5 and MT4.

**Methodology:** The quantification of antibody titer by Fluorescence-activated cell sorter (FACS) was performed as per further description. A single bacterial colony was inoculated in 5ml of LB broth at $37^{o}$ C without shaking. 1ml of the overnight culture was gently pelleted and washed twice with sterile PBS (1% BSA, 0.05% sodium azide). Finally the pellet was resuspended in PBS to attain a bacterial density of $10^{7}$ CFU/ml. The serum sample was diluted to 1:20 in PBS and inactivated at $60^{o}$ C for 30 min. The inactivated serum was centrifuged at 13,000 rpm for 10 min. The supernatant was collected and diluted to 1:20, 1:60 and 1:120.

Similarly for sIgA, the luminal content was flushed with 2 ml of PBS containing 0.05 M EDTA (pH8.0) and 66 mM PMSF. The luminal content was briefly vortexed and centrifuged at 40,000 rpm for 30 min at $4^{o}$ C. The supernatant was collected and used as undiluted, 1:3 and 1:9 dilutions. Further, 25 µl of diluted serum and the gut wash samples were incubated with 25 µl of suspended bacteria for 1 h at $4^{o}$ C. The bacterial cells were washed twice before resuspending in FITC-conjugated monoclonal anti mouse IgG and IgA (Abcam). Then, the cells were incubated for 1 h further at $4^{o}$ C and finally washed once with PBS (1% BSA, 0.05% sodium azide). Subsequently the cells were resuspended in PBS (2% PFA) for acquisition in a FACScanto (BD Biosciences).

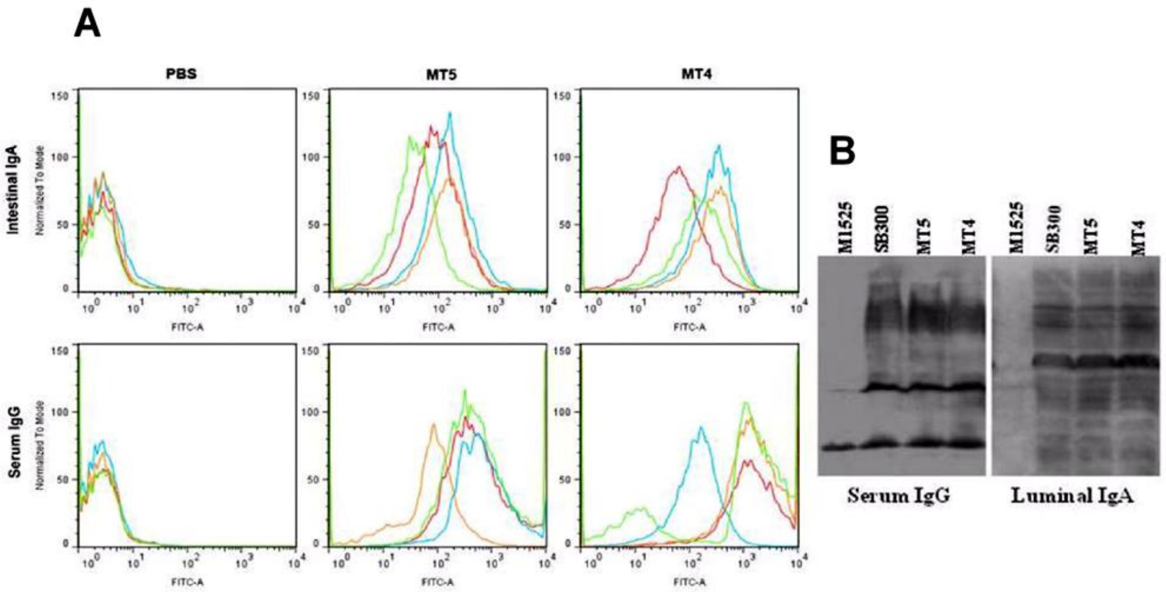


Fig. S4 : Serum and gut wash from mice treated with PBS and vaccinated with MT4 and MT5 were collected, diluted to a highest dilution of 1:120 (serum) and 1:9 (gut wash). The presence of *Salmonella* specific IgG and secretory IgA were detected by bacterial FACS **(A)** and Western blot **(B)**. Each coloured line indicate data obtained from individual mice of respective group. The representative Western blot analysis of the antibody responses was done by developing the blots from the overnight cultures of MT5, MT4, SB300 (wt *S*. Typhimurium) and M1525 (*S*. Enteritidis; negative control) by using the sera and gut luminal sIgA of the immunized mice.

**Analysis:** To validate the immunogenic potency of MT4, the antibody titers from the animals vaccinated with MT4 and MT5 strains was analyzed. Serum and gut wash samples were collected at the end of the day 30 p.v. procedure and antibody responses were quantified via FACS and Western blot analysis. This experiment relies on the specific antibody binding to specific antigens on the surface of the bacterium (wild-type *S*. Typhimurium) as compared to a bacterium of different serovar (*S.* Enteritidis). The intestinal wash and serum samples from mice vaccinated with either MT5 or with MT4 exhibited equivalent responses of *Salmonella* specific serum IgG and luminal secretory IgA.
